# Exploring the Endorsement of Gender Stereotypes and Physical Activity in Young Women

**DOI:** 10.1089/whr.2024.0117

**Published:** 2025-03-17

**Authors:** Melanna Cox, Paige F. Richmond, Annie Shtino, John R. Sirard

**Affiliations:** University of Massachusetts Amherst, Amherst, Massachusetts, USA.

**Keywords:** women, physical activity, self-efficacy, gender norms, sexism, ambivalent sexism

## Abstract

**Background:**

Physical behaviors (PBs), defined as physical activity (PA) and sedentary behavior (SB), are consistently less favorable in women than men. Extensive qualitative research has identified gender norms as a well-known barrier to women’s PA, but they have yet to be clearly conceptualized and quantified. The purpose of this study was to (1) investigate the relationship between benevolent sexism endorsement (BSE) and PB, (2) explore women’s experiences with sexism in PA settings, and (3) identify sexism constructs within focus group discussions.

**Methods:**

Participants completed the Ambivalent Sexism Inventory (0–5 scale) and wore a hip-worn activity monitor for 7 days. Spearman correlations were calculated between BSE and PB. A 90-minute focus group (*n* = 4) was transcribed and coded to identify themes and sexism constructs.

**Results:**

Participants (*n* = 20, 20.7 ± 1.3 years) exceeded PA guidelines and reported low BSE scores (1.8 ± 0.76). Weak associations were found between BSE and objectively measured PA (*r* = −0.19 to *r* = −0.37) and ST (*r* = 0.14). Focus group results yielded four themes: (1) Age-Related Decline in PA, (2) Parental Roles, (3) Peer Relationships/Friendships, and (4) Physical Education Teachers/Coaches.

**Conclusion:**

As hypothesized, associations between BSE and PA outcomes and SB were negative and positive, respectively. Focus group themes were related to benevolent sexism constructs. Future research should be conducted in larger, more diverse samples and consider other factors that may impact one’s endorsement of benevolent sexism.

## Introduction

Strong evidence indicates that men are more physically active than women across the lifespan.^1–3^ In addition, women tend to spend more time in sedentary behavior (SB) than men.^1^ Low physical activity (PA) levels and high SB are associated with poor health outcomes, potentially putting women at a greater risk for reduced health.^4^ Through qualitative studies, perceived gender norms regarding PA participation in women have been well established as a contributor to the PA gender disparity. However, these gender norms regarding PA have yet to be quantified. Therefore, identifying a way to quantify these gender norms from qualitative studies would allow researchers to explore, in larger and more diverse samples, associations and casual mechanisms between women’s experiences and endorsement of gender norms and their participation in PA.

The Social Cognitive Theory (SCT) is historically used to understand PA behaviors and, therefore, can be used to put the many contributors to PA gender disparities in context.^5^ The SCT is a triadic model that emphasizes the reciprocal relationships between environmental, biological, personal, and behavioral factors.^5^ All three factors of the SCT can be observed in perceived gender norms pertaining to PA among women across the lifespan.^6–8^ For example, a small body of research suggests that adolescent girls do not typically participate in physical education classes because they believe that boys are supposed to be more physically active during these classes than girls.^6,7^ The perceived gender norm (personal) that boys should be more active than girls in physical education class (environmental) could lead the girl to make an acute decision to be less physically active (behavioral). Furthermore, the SCT mentions social support (environmental) and self-efficacy (personal), which are two widely accepted psychosocial factors that interact with one another, impacting PA.^5,8^ Social support includes encouragement to be active and being provided with the opportunity to be active, while self-efficacy for PA is one’s confidence to be active. Unfortunately, women consistently report lower perceived social support and self-efficacy for PA suggesting that the disparity in these psychosocial correlates of PA leads to reduced PA behaviors.^8,9^ The SCT provides a strong framework to understand some aspects of gender norms in PA; however, it does not provide insight as to *why* there are gender differences within the theory’s constructs. Therefore, the application of a different theoretical framework may provide insight into why the gender disparity in PA and its psychosocial correlates exist. The ambivalent sexism theory highlights how gender norms and roles negatively impact women in everyday contexts. Furthermore, the Ambivalent Sexism Inventory (ASI) quantifies an individual’s endorsement of sexism. Therefore, unlike the SCT, the ambivalent sexism theory conceptualizes sexism observed in gender norms and quantifies the respondent’s endorsement of sexism, which may provide a better understanding of the qualitative data that exists.^10^

Ambivalent sexism encompasses both hostile and benevolent sexism. Hostile sexism (HSE) is the overt form of sexism in which individuals have a negative view toward women; particularly toward those who go against traditional gender roles.^10^ HSE rarely goes unnoticed and is typically not socially accepted.^10^ Benevolent sexism is a covert form of sexism that includes three constructs: *paternalism* (women should be protected and revered by men), *gender differentiation* (many behaviors/activities are gendered; e.g., “women’s work”), and *heterosexuality* (women are expected to be feminine to attract a stereotypically masculine man). Unlike HSE, benevolent sexism often appears to have positive intentions and frequently goes unnoticed. Despite the positive outward appearance, the implication of benevolent sexism is that women are fragile, incompetent, and do not have the same abilities as men.^10^ Research has shown that both hostile and benevolent sexism have negative effects on women such as decreases in occupational, academic, and cognitive performance.^11,12^ Intrusive thoughts of incompetence, which are less overt when compared with the psychological damage of HSE, are believed to be the primary mechanism by which benevolent sexism adversely affects women.^11–13^ Competency is described as the extent to which women feel they possess the capabilities needed to complete a task and is developed through experiences over time.^14^ Although different, competency and self-efficacy are closely related in that competency is necessary to develop self-efficacy.^14^ The connection between competency and self-efficacy demonstrates the direct parallels between how gender roles can decrease a woman’s competency in life tasks and how gender roles can decrease a women’s PA through insufficient self-efficacy.^15–17^ Despite these theoretical similarities, past studies have yet to evaluate how benevolent sexism endorsement (BSE) and experiences may be related to a woman’s PA behavior.

Therefore, the purposes of this exploratory mixed-methods study were to (1) investigate the endorsement of benevolent sexism as a potential correlate of objectively assessed PA and SB in young women, (2) use focus group data to understand women’s experiences with and endorsement of benevolent sexism within PA contexts, and (3) identify benevolent sexism constructs within focus group results.

## Methods

### Study design

This exploratory mixed-methods study included quantitative PA (1 week of wearable accelerometer), benevolent and HSE measures, and demographic questions, in addition to qualitative data (focus group) regarding attitudes and opinions about gender norms in PA.

### Participants

Participants were recruited via flyers and word of mouth on the University of Massachusetts Amherst campus. Participants (*n* = 20) underwent screening for study eligibility at the Physical Activity and Health Laboratory at the University of Massachusetts Amherst. Participants had to be college women between 18 and 24 years old, having no physical or cognitive limitations that could hinder their ability to participate in PA or complete the study. An institutional review board (IRB)–approved informed consent was obtained by trained research staff once eligibility was determined. All procedures in this study were approved by the University of Massachusetts Amherst IRB.

### Quantitative measures

#### Ambivalent sexism

**FIG. 1.**
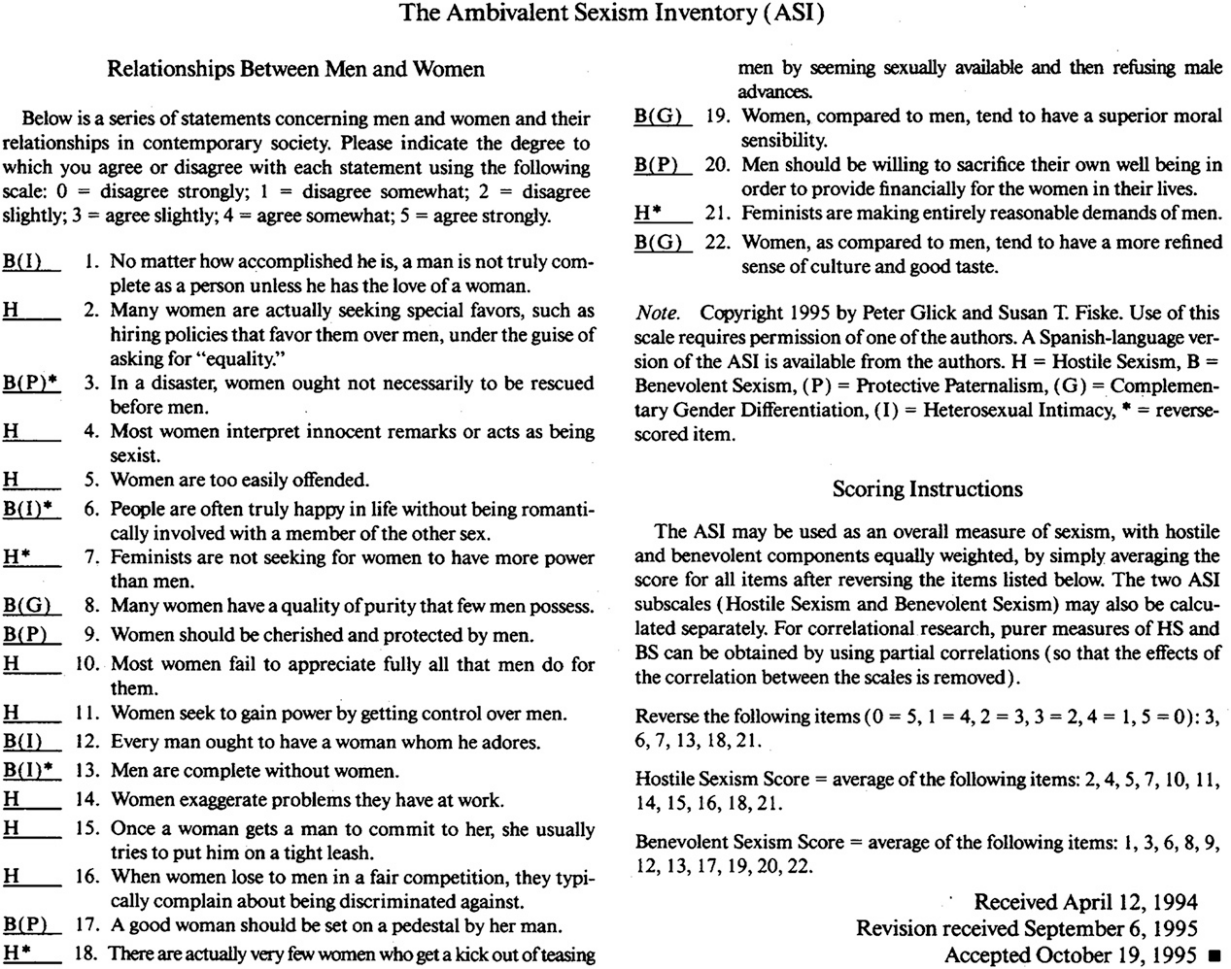
Ambivalent Sexism Inventory by Glick and Fiske.^10^ Copyright 1996, Glick and Fiske. Reproduced by permission.

The ASI is a 22-item questionnaire that measures a person’s endorsement of hostile (11 items) and benevolent (11 items) sexism.^10^ The 11 benevolent sexism items are further categorized by the three subscales: gender differentiation (3 items), paternalism (4), and heterosexuality (4 items). Participants ranked their level of endorsement for each statement using a 5-point Likert scale, from “strongly disagree” (1) to “strongly agree” (5). Per the recommendation from Glick and Fiske, responses from specific items on each subscale were reverse coded and averaged independently (Fig. 1).^10^ Although the focus of the study is benevolent sexism, HSE was included for the purpose of clarity.

#### Physical activity

The ActiGraph GT9X Link (ActiGraph, Pensacola, FL), a small wearable accelerometer that records and stores raw acceleration data, was used to collect objective PA data. The device can be initialized to sample between 30 and 100 Hz using ActiLife software (version 6.13.3); 80 Hz was used for this study. ActiGraph devices were placed on the right hip using manufacturer-provided adjustable elastic belts. Non-wear time was calculated using the Choi algorithm.^18^ The final dataset only included participants with at least four valid wear days (at least 10 hours of wear time per day).

The Freedson 1998 cutpoints in the ActiLife software were used to calculate average time per day spent in SB, light (LPA), moderate (MPA), vigorous (VPA), and moderate-to-vigorous PA (MVPA). Metabolic Equivalents of Task (MET)-minutes per day from the accelerometer data were also calculated.^19^ A MET is an absolute intensity value based on 1.0 MET being equal to resting metabolic rate; a 4.0 MET activity would require 4.0 times greater than resting metabolic rate. Minutes spent in MPA and VPA were multiplied by 3.5 and 8.0 METs, respectively, to calculate MET-minutes from accelerometer data, consistent with commonly used ranges for SB (≤1.5 METs), LPA (1.5–2.9 METSs), MPA (3.0–5.9 METs), and VPA (≥6.0 METs).^20^

#### Anthropometrics and demographics

A trained female research assistant measured weight and height using a research-grade scale (SECA, Hamburg) and a portable stadiometer (Weigh and measure, LLC, Olney, MD). Weight was measured to the nearest 0.1 kg and height to the nearest 0.1 cm. Participants self-reported age, race, occupation, education level, gender identity, and sexual orientation.

### Study procedures

After consent was obtained, participants completed the demographic questionnaire, and height and weight were measured. Participants were then instructed to wear the hip-worn ActiGraph GT9X Link for seven consecutive days during all waking hours except when the device would get completely wet (showering, bathing, swimming). The device was secured to an appropriate-sized band, and researchers demonstrated how to wear and adjust the band if needed. Participants left the lab wearing their device, which was set to begin collecting data the following day at 04:00 AM. The participants returned the device after seven full consecutive days of wear. The data were downloaded using the ActiLife software on a secure laboratory computer, and summary statistics were calculated.

### Quantitative data analysis

Descriptive statistics for the accelerometer data were computed, indicating that several variables were positively skewed. Therefore, non-parametric statistics were used for all analyses. Spearman correlation coefficients between ASI scores and PA outcomes were calculated. Calculated correlation coefficients were classified as very weak (|<0.2|), weak (|0.20–0.40|), moderate (|>0.40–0.60|), and strong (>|0.60|) per recommended classifications from Evans.^21^ All statistical analyses were performed in R-software (version 3.4.1).

### Focus group procedures

#### Participant selection

Participants were randomly selected and invited to participate in the 1.5-hour focus group to include focus group participants with various physical behaviors (PBs). After three rounds of randomization (due to loss to follow-up), six participants were invited to the focus group, but only four participants attended the focus group session.

#### Focus group structure

The focus group was semi-structured and aimed to assess if sexist constructs could be observed without directly asking about sexism. Therefore, the focus group questions were organized to first explore their past and present experiences with PA and broader reflection questions related to women and PA (Table 1). However, the moderator asked participants follow-up questions that may not have been a part of, or in the same order as, the *a priori* questions.

**Table 1.** Focus Group Questions

Category	Questions
History	What was your family dynamic growing up? (single parent/siblings/etc.) What did you do for fun growing up? What kind of activities did your parents encourage you to do? What was your first memory of being active? Did you have recess? What was your experience in physical education classes? *What kinds of activities were offered to you?* *What did you do or like doing?* *Did you have school/gym uniforms?* Did you play sports? *What sports did you play AND/OR did you want to play?* How do you think these childhood/adolescence years impacted your current physical activity?
Present	What do you do currently to be active? How comfortable are you with being active? What would make being active easier?
Reflection	What experiences do you have with all-women’s PA activities? What experiences do you have with non-gendered PA activities? How would you describe the experience of women in physical activity?

One female researcher moderated the focus group, and a second female researcher observed and took notes, including body language or tone of voice. The focus group was recorded using an audio recorder. Upon arriving, participants were reminded of the purpose of the focus group and asked if they had any questions or concerns. The moderator instructed the participants to select a pseudonym to protect their identity. Pseudonyms were later used to identify speakers in the transcript. Participants were asked to avoid using any real names or locations that could identify any participants. The audio recorder was started once all questions were answered and pseudonyms were selected.

The audio from the focus group was processed through an automatic transcription software (Otter.ai, Los Altos, CA) to produce a raw transcript. Two researchers independently listened to the audio, corrected any automated transcript errors, and identified speakers. A third researcher listened to the audio while reading both transcriptions in tandem to assure accuracy. A final transcription was created and used for all qualitative analyses.

### Focus group analysis

Researchers coded the transcript using open coding to identify any emerging themes. Two researchers read through the transcript independently and identified meaningful quotes. Each researcher organized the quotes into overarching themes. The researchers then compared their quotes and themes. Once a consensus was reached regarding the themes and respective quotes, the researchers re-read the transcript to extract more quotes that may fit into the designated themes. The most representative quotes from each theme were highlighted. The quotes were then labeled as a form of BSE or HSE, whether the quote endorsed or refuted the sexism construct. For example, if a participant stated that their parents encouraged them to participate in stereotypical “boy” sports, that quote would be labeled as, “benevolent sexism-gender differentiation” (e.g., BSE-GD) despite the tone of the comment being in opposition of the connotation of the construct. Quotes labeled as benevolent sexism were further labeled by the constructs, paternalism, gender differentiation, or heterosexuality. Any notable remarks made about past experiences during this focus group were highlighted and referenced. Quotes for each participant were categorized as positive or negative experiences because it is expected that more positive experiences would elicit more PA. The positive quotes were experiences that encouraged PA and/or elicited a positive emotion in the participant and negative quotes were experiences that discouraged, hindered and/or elicited a negative emotion from the participant. Bar charts were created to graphically represent each focus group participant’s BSE and HSE scores and selected PA data to better observe patterns across the four focus group participants.

## Results

All participants identified as a woman and were college students (20.7 ± 1.3 years). The sample was majority white and identified as heterosexual. On average, this sample had a healthy body mass index (BMI). Most participants were either in the process of completing their bachelor’s degree or had recently obtained it (Table 2).

**Table 2.** Participant Demographics (*n* = 20)

Demographic	Mean ± SD or %
Age (years)	20.7 ± 1.3
BMI	27.3 ± 8.1
Gender (% women)	100
Race (%)	
White (*n* = 18)	80
Black/African American (*n* = 2)	10
White/Hispanic/Latino (*n* = 2)	10
Sexuality (%)	
Heterosexual (*n* = 16)	80
Asexual (*n* = 1)	5
Bi-sexual (*n* = 1)	5
No response (*n* = 2)	10
Education (%)	
Bachelor’s degree in progress (*n* = 17)	85
Bachelor’s degree completed (*n* = 2)	10
Master’s degree completed (*n* = 1)	0.5

BMI, body mass index.

### Physical behaviors and ambivalent sexism scores

Participants were mostly active and reported low BSE and HSE scores (Table 3). Five participants were not included in the data analysis due to loss of device or insignificant wear time (*n* = 15) (Table 3). The participants were accumulating approximately 60 minutes of MVPA per day (median = 57.4 minutes/day, interquartile range [IQR] = 36.7). Notably, VPA was negligible (median = 0.6 minutes/day, IQR = 3.4), and therefore, minutes in MVPA were mostly comprised of MPA. Participants were sedentary for roughly 8.5 hours per day (median = 8.7 hours/day, IQR = 1.9). Participants reported both low BSE (median = 1.14, IQR = 0.91) and HSE scores (median = 0.54, IQR = 1.07). The HSE scores were slightly lower than the BSE scores.

**Table 3.** Descriptive Statistics: Daily Minutes of Physical Activity and Ambivalent Sexism Inventory Scores

PB outcomes (*n* = 15)	Mean	Std dev	Median	Lower quartile	Upper quartile	Interquartile range	Min	Max
Sedentary behavior	536.0	83.5	524.0	465.0	582.3	117.3	441.5	779.1
Light PA	207.2	49.1	204.8	171.7	247.4	75.7	139.0	313.4
Moderate PA	52.4	19.7	55.2	30.5	66.0	35.5	23.8	87.8
Vigorous PA	1.8	2.3	0.6	0.0	3.4	3.4	0.0	6.7
Moderate-Vigorous PA	54.1	21.2	57.4	30.5	67.2	36.7	23.8	91.3
Moderate MET-mins	183.2	69.0	193.1	106.8	230.9	124.1	83.1	307.4
Vigorous MET-mins	14.0	18.2	4.5	0.0	27.0	27.0	0.0	53.3
Total MET-mins	197.3	81.7	216.3	106.8	246.3	139.6	83.1	335.4
ASI scores								
Benevolent sexism	1.80	0.76	1.14	1.13	2.04	0.91	0.50	3.4
Hostile sexism	0.82	0.70	0.54	0.36	1.43	1.07	0.00	2.18

ASI, Ambivalent Sexism Inventory; MET, Metabolic Equivalents of Task; PA, physical activity.

### Associations between ambivalent sexism scores and physical behaviors

A weak positive association was found between BSE and SB (*r* = 0.14). Weak negative associations were found between BSE and estimates of average daily LPA, MPA, VPA, MVPA (*r* = −0.37 to −0.18) and average daily hours of MPA MET-minutes, VPA MET-minutes, and total MET-minutes (*r* = −0.37 to −0.33). The associations between HSE scores were lower than associations between BSE and PA outcomes. A weak negative association was found between HSE and SB (*r* = −0.22). No associations were found between HSE and PA outcomes (*r* = −0.03 to 0.05).

### Focus group participants

#### Demographics

Participants (*n* = 4) who participated in the focus group were undergraduate (*n* = 3) and graduate (*n* = 1) students in their early 20s (21.8 + 0.9), mostly White, heterosexual, with normal BMIs, excluding one participant who was classified as obese (BMI = 30.3 + 10.9).

#### Ambivalent sexism scores and physical behaviors of focus group participants

**FIG. 2.**
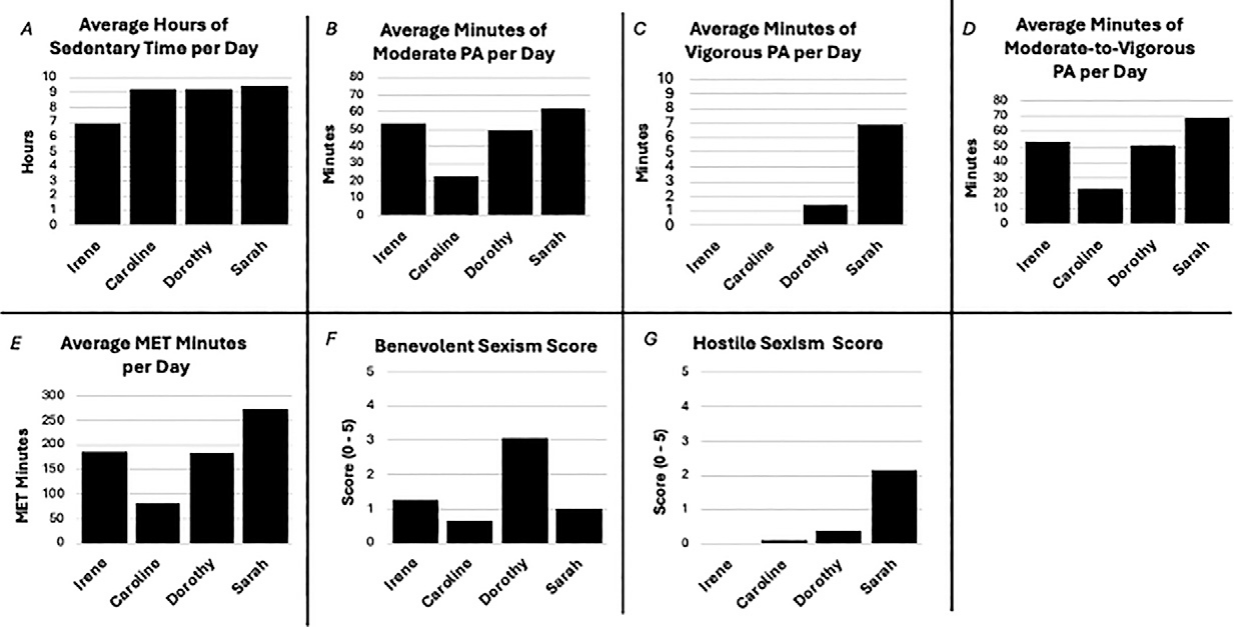
Focus group participants’ endorsement of ambivalent sexism and physical behaviors.

The bar charts (Fig. 2) graphically represent each participant’s BSE and HSE scores, average minutes per day of PA and SB. The participants’ BSE scores ranged from 0.63 to 3.09) on the 5-point scale. Caroline (0.63), Sarah (1.00), and Irene (1.20) had notably lower BSE scores than Dorothy (3.09). The participants’ HSE scores were nearly non-existent for three participants (0.0–0.36). Caroline (0.0), Irene (0.0), and Dorothy (0.36) had notably lower HSE scores than Sarah (2.1). All focus group participants met PA guidelines of 150 minutes of MVPA per week, ranging from 23 minutes/day (Caroline) to 69 minutes/day (Sarah) of MVPA (Fig. 2D). On average, Irene was the least sedentary (∼7 hours). The remaining participants accumulated approximately 9 hours of sedentary time each day (Fig. 2A). Despite Dorothy’s notably higher BSE score, compared with the other participants, she was just as active as Irene (∼50 minutes of MVPA/day in Fig. 2D) and as sedentary as Caroline and Sarah (∼9 hours of sedentary time/day in Fig. 2A).

#### Focus group results

The focus group results included four themes: age-related decline in pa, parental roles, peer relationships and friendships, and physical education teachers and coaches. Table 4 contains the theme definitions, the exemplary quotes expressing both positive and negative aspects of the themes, and categorization based on sexism construct. Among the total number of quotes identified (*n* = 38), the number of positive (19 quotes) and negative experiences (19 quotes) were the same. Although paternalism was present, gender differentiation was the prominent benevolent sexism construct observed across all quotes and was found in both positive and negative experiences. No clear examples of heterosexuality were observed. In addition, negative experiences included four HSE quotes. Participants expressed the most positive experiences with peers and friends and the most negative experiences with physical education teachers and coaches.

**Table 4.** Focus Group Themes and Quotations

Theme/term	Definition	Positive experience	Negative experience
Age-related decline in PA	Change in PA levels as the participants aged.	“… I did dance for like five years, like competitively and then I did softball and golf too.”—**Dorothy** (on what she did as a child) *BSE-GD* “I didn’t like sports as a kid and then when I hit Middle School, that’s when I started caring”—**Irene** “I did like every typical kid sport stuck with soccer for the longest”—**Caroline**	“But like then when high school was over then I would like, do like, intramurals like once here but that was about it. And like go to the gym sometimes.”—**Dorothy** “…when I was younger, I was all about like, nature, documentaries and science.”—**Irene** *BSE-GD* “In high school, that, [recess] was like, kind of a time where you’d like catch up between classes and get like a little gossip before you had to go like to third fourth period. So it’s basically like get a snack, go to the bathroom BSE-GD…”—**Irene** “In undergrad, I would go to like the actual gym more than now because I don’t really have the time”—**Dorothy** “… I usually get home late. And I’m just like, drained. And when I do have free time, I’m like, I just want to just like decompress.”—**Irene**
Parental support	The father’s and mother’s opinion/role in the participants’ PA levels as children.	“My family, mostly my dad, my mom’s a little bit more laid back, but he really wanted me to just try everything out.”—**Caroline** *BSE-GD* “…he would drive me to dance. But like yeah he’d always like play catch or like throw like if I wanted to like go hitting like he’d bring us to the park and then he would bring us golfing.”—**Dorothy** *BSE-GD* “…she would try…she would try, but she would always go to like our games and stuff and any competition I had.”—**Dorothy** “…my mom liked me swimming, so that was like interesting.”**—Sarah** *BSE-GD* “…my mom’s a little bit more laid back.”—**Caroline** “And my mom was really great about like, being like, it [body image] shouldn’t matter…”—**Caroline** “So, she wasn’t like a huge sports person. Like if I showed interest, she’d encourage it.”—**Irene** *BSE-GD*	“But my dad didn’t like me swimming… he wanted me to be an artist.”**—Sarah** *BSE-GD* “My mom, she’s the quintessential like I would say girly girl, so she never cared about sports…but she wasn’t like pushing like you need to get your hour a day of running or activity. She was like you’re fine…So, If I wanted to do sports, I would usually hang out with my aunt…”—**Irene** *BSE-GD* “…but she, when I was young like I was already throwing it like too hard for her. She’s not that athletic.”**—Dorothy** *BSE-GD*
Social support: peer relationships and friendships	How peer relationships/friendships influenced the participants’ PA levels and activities.	“I prefer to work out with like a lot of my friends…”**—Irene** “We [athletes] have like our own gym and like lifting place…like everybody’s there…so it’s like it’s like fun and there’s like loud music and so like, it’s like fun and people get into it.”**—Sarah** “I remember senior year I wanted to do tennis because my friends were doing it…”**—Dorothy** “…and all my friends are like, ‘let’s try out for volleyball.’ I was like, this seems dumb but okay.”—**Irene** *BSE-GD* “Like, I loved playing kickball in elementary school. So me and my friends would always, like get a group together like oh we’re playing today. So that was like my main thing I loved.”—**Irene** “…okay, we’re playing with just girls today it’s going down…”**—Irene** *BSE-GD* “…this summer me and my brothers are going to do biking because there is a bunch of like trails around here..”**—Dorothy**	“…I’d always wanted to do track in the offseason. And all of my friends were like ‘the nerds do track, don’t do track’ and I was like, Okay, I guess I won’t do it…”**—Irene**
Social support: physical education teachers and coaches	How the participants’ relationships with their physical education teachers/classes and coaches influenced their PA levels.	“But guy coaches loved me like, we would like to get along, and they’d be able to coach me well.”—**Sarah** “When I played club [sports] I had a female coach, and that was amazing.”**—Irene**	“It was very discouraging. I’d say in some situations when I feel like a lot of teachers would highlight the males in their performance and other people, even some of the boys who aren’t as quote-unquote athletic kind of like, all right, I’m not going to try then because I know I can’t beat them.”**—Irene** *BSE-GD* “…it depends on your coach to train that competitive competitiveness to something positive. So my coach wasn’t very good about that…”**—Irene** “He [her coach] was very notorious for saying ‘I hate coaching girls’…he was very angry and so he would have a bad habit of getting like red flags and games to like break his clipboard over his knee because he gets so frustrated he’d walk out the the gym…”**—Irene** *HSE* “…I was putting my hair in a bun before I was putting my cap on, and she [her coach] was like, ‘This isn’t a fashion show. Hurry up.’”**—Sarah** *HSE* “So they’d [physical education teachers] be like, do your requirement, and then you could just sit in the bleachers. And they didn’t care. You could sit there until the hour was over.”**—Irene** *BSE-GD* “Yeah, and just like he [her coach] you could just tell his patience level with us was much lower than the guys even though honestly the guys like messed around so much more than us and we won the division, and they didn’t…”**—Irene** *HSE* “Like I think it depended which quarter you had the teachers they [physical education teachers] lost steam by the fourth one they didn’t really care.”**—Caroline** “I would say that they [physical education teachers] focused ironically, on the people who didn’t need as much like focus like they focus on people who were like athletically inclined or doing well rather than the kids who were kind of falling into the back and fall back a little bit.”**—Irene** “So all the boys in my grade played soccer, and our gym coach was the soccer coach, so he was always like, yeah, to them, and we were just kinda like running back and forth so whatever.”—**Dorothy** *BSE-GD* “I actually like when I decided that I wasn’t going to go to school that only had a woman’s team because of like, I’m just like, there’s so much more drama.”**—Sarah** *HSE*

BSE-GD, benevolent sexism endorsement–gender differentiation; HSE, hostile sexism.

### Themes

#### Age-related decline in PA

Age-related decline in PA was defined as change in PA levels as the participants aged. The participants shared similar lifelong changes in PA participation. Reflecting on their childhood, participants mentioned being active through various sports. For example, Caroline stated, “I did like every typical kid sport and stuck with soccer for the longest.” After high school, participants mentioned time and energy being a barrier to their PA. Irene stated, “… I usually get home late. And I’m just like, drained. And when I do have free time, I’m like, I just want to decompress.” Notably, Sarah was a recently retired collegiate athlete and, therefore, did not express the same decline seen in the other participants’ experiences. In fact, she was the most active participant for each PA outcome. Age-related Decline in PA was unique because it was the only theme where participants did not mention other individuals as a contributor to their PA participation.

#### Parental roles

Parental roles were defined as a father’s and/or mother’s opinion/role in the participant’s PA participation. The participants’ experience with their father regarding PA varied but all participants shared the same sentiment that their mothers were encouraging regarding PA. Caroline said, “My family, mostly my dad, my mom’s a little bit more laid back, but he really wanted me to just try everything out.” Dorothy expressed a similar experience when she said, “…when I was young like I was already throwing it like too hard for her. She’s not that athletic…but she would always go to like our games and stuff and any competition I had.” Furthermore, Dorothy appeared amused by her mother’s lack of athleticism but also appreciative of her support. Although Irene did not grow up with her father, she did still share a similar sentiment about her mother as did Dorothy. Also appearing amused but appreciative, she said, “My mom, she’s the quintessential like I would say girly girl, so she never cared about sports…but she wasn’t like pushing like you need to get your hour a day of running or activity. She was like you’re fine… So, if I wanted to do sports I had to hang out with my aunt.” She added, “So, she [her mother] wasn’t like a huge sports person. Like if I showed interest, she’d encourage it.” Sarah described slightly different experiences with her parents. She shared that her father did not support her involvement in swimming. She stated, “But my dad didn’t like me swimming…he wanted me to be an artist.” However, she did follow up and said, “…my mom liked me swimming, so that was like interesting.” Sarah appeared slightly amused by her defiance when mentioning her father’s thoughts on swimming. In contrast, her tone when she spoke of her mother’s support showed genuine consideration to why her mother would support her despite her father’s lack of support. Importantly, gender differentiation was challenged in the cases of all participants excluding Sarah. The remaining participants’ parents supported and encouraged any activity rather than directing them to solely “girl” activities. However, Sarah’s father discouraged swimming and encouraged art which would perpetuate traditional gender differentiation.

#### Peer relationships and friendships

The peer relationships and friendships theme was defined as how peer relationships and friendships influence the participants’ PA participation and activities. The experiences with peers conveyed by the participants centered around their conscious decisions to be active or not. For example, Irene said, “…I’d always wanted to do track in the offseason. And all of my friends were like ‘the nerds do track, don’t do track’ and I was like, Okay, I guess I won’t do it…” In comparison, Dorothy decided to participate in a sport because of her friends. She said, “I remember senior year I wanted to do tennis because my friends were doing it…” Although Irene decided not to participate in track, her tone showed no sign of disappointment but rather enthusiasm like that of Dorothy’s who did decide to be active.

#### Physical education teachers and coaches

The physical education teachers and coaches theme was defined as how the participants’ relationships with their physical education teachers/classes and coaches influenced their PA participation. The participants’ experiences with physical education teachers and coaches were mostly negative. Their experiences in physical education class focused on feelings of being ignored by teachers which led them to not want to participate. Irene sounded slightly frustrated when she said, “It [physical education class] was very discouraging. I’d say in some situations when I feel like a lot of teachers would highlight the males in their performance and other people, even some of the boys who aren’t as quote-unquote athletic kind of like, all right, I’m not going to try then because I know I can’t beat them.” Furthermore, Dorothy experienced the lack of attention even in cases where they [females] were involved in the activity. She said, “So all the boys in my grade played soccer, and our gym coach was the soccer coach, so he was always like, yeah, to them, and we were just kinda like running back and forth so whatever.” Interestingly, Dorothy did not seem outwardly bothered by this sentiment. Even if the teachers did not intend harm, both experiences demonstrate gender differentiation in which boys can be challenged physically but girls cannot and/or not considered to provided time and attention to encourage PA. As for experiences with coaches, Irene and Sarah appeared to have the most experience with coaches and shared clear instances of HSE but perceived them quite differently. Both participants spoke strongly about the gender of their coaches. Irene expressed her strong disdain for her negative experience with HSE when she was coached by a man. She said, “He [her coach] was very notorious for saying ‘I hate coaching girls’…he was very angry and so he would have a bad habit of getting like red flags and games to like break his clipboard over his knee because he gets so frustrated he’d walk out the gym…” Sarah expressed similar experiences with a male coach but her perception of the experience was different from Irene’s. She was visibly excited and enthusiastic when she said, “I actually like when I decided that I wasn’t going to go to school that only had a woman’s team because of like, I’m just like, there’s so much more drama.” She also expressed experiences with HSE when she said, “…I was putting my hair in a bun before I was putting my cap on, and she [her coach] was like, ‘This isn’t a fashion show. Hurry up.’” Despite the overtly sexist comment, she mentioned that male coaches “…loved her.” Her more positive perception of these hostile actions coincides with her HSE score that was noticeably higher than the other three participants.

## Discussion

The purpose of this exploratory mixed-methods study was to assess the relationship between benevolent sexism and PBs in young women and to identify if a focus group discussion would elicit quotes that represent benevolent sexism constructs. In this small sample of young adult women, we observed weak associations between BSE scores and objectively measured PBs and primarily no associations between HSE scores and PBs. However, these associations were in the hypothesized directions (positive associations with SB and negative associations with PA). The focus group results revealed themes consistent with the literature and encompassed sexist constructs. The nature and perception of the experiences related to those themes varied among participants. To our knowledge, this is the first study to assess the relationship between benevolent sexism and PBs, with the results indicating a need for continued research to explore these associations and causal effects.

The current study observed weak negative and positive associations between BSE scores and PA and SB, respectively. Although this is the first study to assess the relationship between benevolent sexism and PBs, the results of this study show a similar relationship in which the endorsement of benevolent sexism has a negative effect on a given outcome.^10–13,22^ For example, King et al. conducted a study to assess male and female managers’ work opportunities, promotions, and responsibilities.^22^ The results revealed that endorsement of benevolent sexism is negatively related to opportunities presented to women despite women and men expressing equal interests in more challenging opportunities.^22^

There were no associations found between HSE scores and PA outcomes and therefore, it is difficult to reach a conclusion with certainty. A small negative relationship between HSE scores and SB was observed. One of the focus group participants (Sarah) had one of the highest HSE scores among all participants in the full sample. Sarah was a previous collegiate athlete whose father did not support her participating in sports and, instead, wanted her to be an artist. Although it may be counterintuitive, past research has demonstrated that exposure to HSE can improve women’s performance for certain outcomes.^11–13^ One study assessed the acute effects of experiencing hostile or benevolent sexism on problem-solving tasks. The researchers found that women exposed to HSE improved their performance during problem-solving tasks than those exposed to benevolent sexism.^23,24^ However, the endorsement of and experience with HSE is not an appropriate approach to increasing PA or decreasing SB in women because it perpetuates existing gender inequalities and has other negative outcomes.^10,23–27^

As expected, focus group data revealed themes that were consistent with the literature and benevolent sexism constructs. Unsurprisingly, much of the discussion was related to social support. First, as supported by previous research, participants discussed how they consciously chose to participate in physical activities based on the decisions of their peers, particularly during middle school and high school.^28^ Also widely known, the focus group participants shared a substantial amount of information that suggested parental support for activity was important during their youth. However, an interesting pattern observed was that all participants described their mother as encouraging but passive in their PA participation while their fathers were more involved in those activities. This observation is notable because it appears to align with traditional gender differentiation ideals such as women being better at inactive, interpersonal skills (emotional support, encouragement) while men are better at physical activities (skill development, instruction). Future research could explore this by comparing women’s experiences with PA with their parents to their parent’s PBs and endorsement of sexism.

Some contradictions were observed between focus group data and women’s sexism scores. For example, two of the women’s perceptions of their mothers identified a form of paternalism and gender differentiation. Dorothy and Irene, who had similar PA levels, said that their mothers were inactive suggesting that both Irene and Dorothy should report similar benevolent sexism scores. However, their benevolent sexism scores were substantially different, which may be attributed to the different benevolent sexism constructs that applied to the women’s statements. First, Dorothy expressed multiple experiences with being active with her father, specifically, because her mother was not athletic enough. Her paternalistic view of her mother’s capabilities is reflected in her high benevolent sexism score. In contrast, Irene spoke about her mother being inactive, not because of her mother’s athleticism but because she identified her mother as “girly” and uninterested in sports. Irene’s statement covertly captures gender differentiation because she highlights her mother’s disinterest in sports by preemptively mentioning her outwardly feminine experience implying that the two are connected. In contrast, Dorothy overtly stated that her mother was *incapable* of catching a ball because she threw it too hard. Her overt mention of paternalism may be why it is reflected in her score but not in Irene’s. Lastly, the general focus group questions did not appear to elicit information regarding heterosexuality (one of the three benevolent sexism constructs), which will require further research to better understand this aspect of benevolent sexism on young women’s PBs.

## Strengths and Limitations

This exploratory study has several strengths and limitations. A significant strength of this interdisciplinary study is the application of the ambivalent sexism theory within the PBs domain, which goes beyond the surface of established, yet not well-understood factors related to PA gender disparities. PBs were measured with a widely used ActiGraph wearable research accelerometer.^29^ Endorsement of sexism constructs was measured using the ASI, which has been validated in several populations and used in various areas of sexism research.^10–12,22,30,31^ Additionally, the mixed-methods design allowed for the application of the ambivalent sexism theory to direct quotes regarding barriers to PA. There were also limitations to the current study. First, the sample size was small, and some recently identified correlates of BSE were not collected on the survey.^32^ Current research suggests that people who are Black, politically conservative, or follow Christian religions are more likely to endorse benevolent sexism than others.^32^ Therefore, future work should account for these demographics when assessing BSE and PBs. Secondly, the range (0.5–3.4) and average (1.8 ± 0.76) of benevolent sexism scores in this sample were relatively low despite observing sexist constructs in the focus group data. It is possible that this discrepancy between scores and statements made during focus groups could be that the ASI is not capturing modern examples of benevolent sexism. For example, the ASI asks, “A good woman should be set on a pedestal by her man.” A modern example of this would be the increasing need for those in relationships to see their partner sharing their relationship on social media.^33^ Moving forward, researchers should consider revisiting the wording and psychometric properties of the ASI. Despite the small, relatively homogeneous sample and low associations, the associations were in the hypothesized directions and sexist constructs were observed in the focus group data.

## Conclusion and Future Directions

This exploratory study found weak associations in the hypothesized directions between endorsement of benevolent sexism and PA and SB. Well established themes such as parental influences, social support outside the home, and PA experiences (gleaned from the focus group discussion) related to benevolent sexism constructs were observed and differed among participants. Studies with larger and more diverse samples plus more in-depth focus group questions and one-on-one interviews will provide more information regarding how each construct of benevolent sexism is related to women’s PA and SB. Lastly, the authors recognize that gender disparities begin early in life and therefore encourage studies to explore this topic in adolescent girls.^2,28,34,35^ This novel area of research should be continued to conceptualize factors of gender disparities in PB to dismantle negative normative beliefs and gender discriminatory practices and policies.

## Abbreviations Used

ASI: Ambivalent Sexism Inventory
BMI: Body Mass Index
BSE: Benevolent Sexism Endorsement
BSE-GD: Benevolent Sexism Endorsement-Gender Differentiation
HSE: Hostile Sexism Endorsement
LPA: Light Physical Activity
METS: Metabolic Equivalents of Task
MPA: Moderate Physical Activity
MVPA: Moderate-to-vigorous Physical Activity
PA: Physical Activity
PB: Physical Behaviors
SB: Sedentary Behavior
SCT: Social Cognitive Theory
VPA: Vigorous Physical Activity
